# Supplementary material for: Decoding DNA sequence-driven evolution of the human brain epigenome at cellular resolution
Source: bioRxiv. 2023 Sep 17:2023.09.14.557820. Preprint. [Version 1] doi: 10.1101/2023.09.14.557820 (PMC10515917; doi:10.1101/2023.09.14.557820)
Supplement: Supplement 1 [file NIHPP2023.09.14.557820v1-supplement-1.pdf]

bioRxiv preprint doi: https://doi.org/10.1101/2023.09.14.557820; this version posted September 17, 2023. The copyright holder for this preprint (which was not certified by peer review) is the author/funder, who has granted bioRxiv a license to display the preprint in perpetuity. It is made available under aCC-BY 4.0 International license.

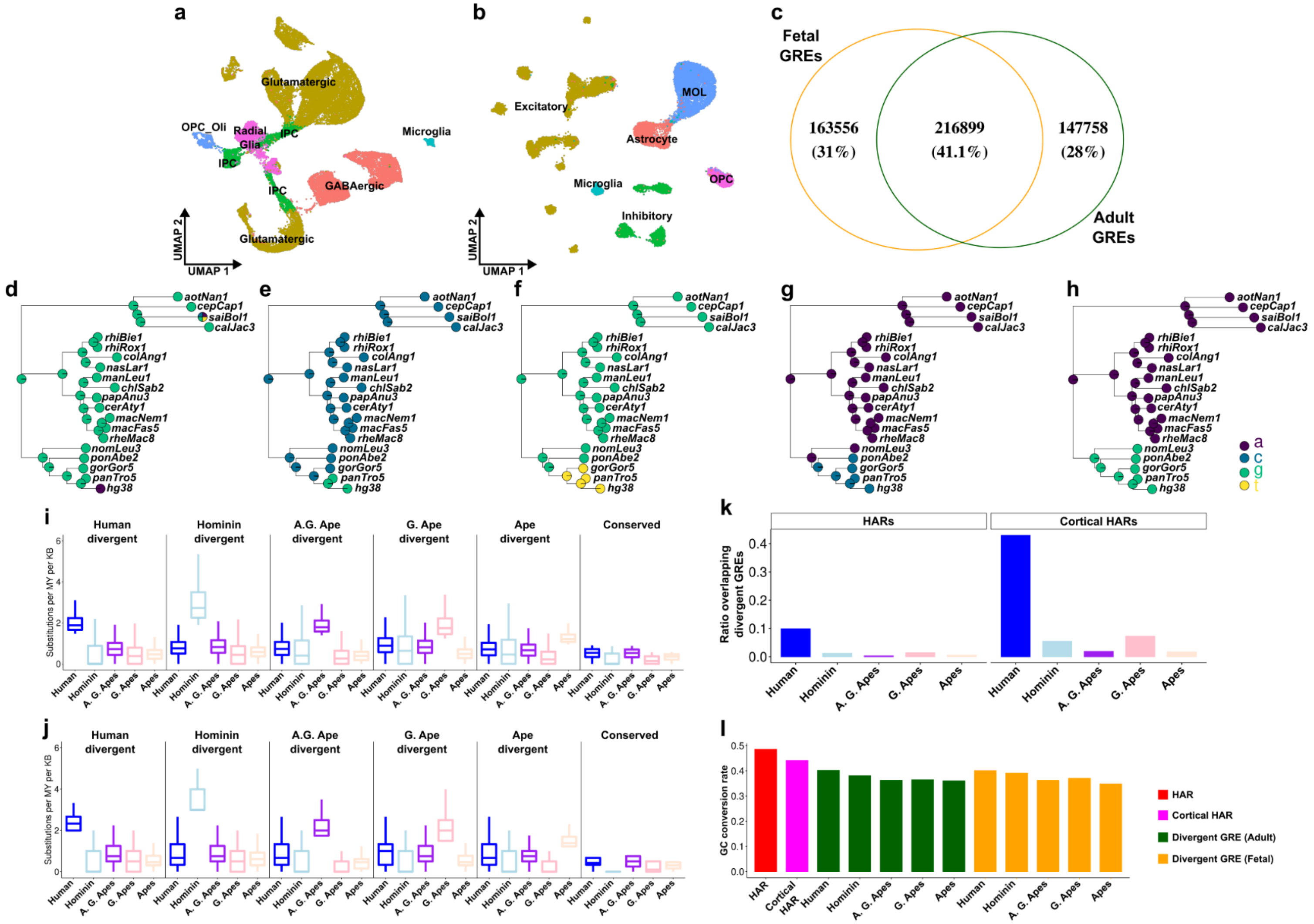

Supplementary Figure 1: Additional plots on the datasets, substitutions and divergent GREs. (a-b) UMAP plots of fetal **(a)** and adult **(b)** datasets. **(c)** Overlap of GREs between adult and fetal datasets. **(d-h)** Phylogenies depicting lineage-specific substitutions in human **(d)**, hominin **(e)**, A.G. Ape **(f)**, G. Ape **(g)** and ape **(h)** lineages. Circles correspond to species included in the dataset and ancestral nodes with a reconstructed sequence. **(i-j)** Boxplots of substitutions per million year per kb for all lineage-divergent GREs and conserved GREs. **(k)** Ratio of HARs / cortical HARs overlapping divergent GREs. **(l)** Ratio of GC conversion in HARs, cortical HARs and lineage-divergent GREs in adult and fetal datasets.

bioRxiv preprint doi: https://doi.org/10.1101/2023.09.14.557820; this version posted September 17, 2023. The copyright holder for this preprint (which was not certified by peer review) is the author/funder, who has granted bioRxiv a license to display the preprint in perpetuity. It is made available under aCC-BY 4.0 International license.

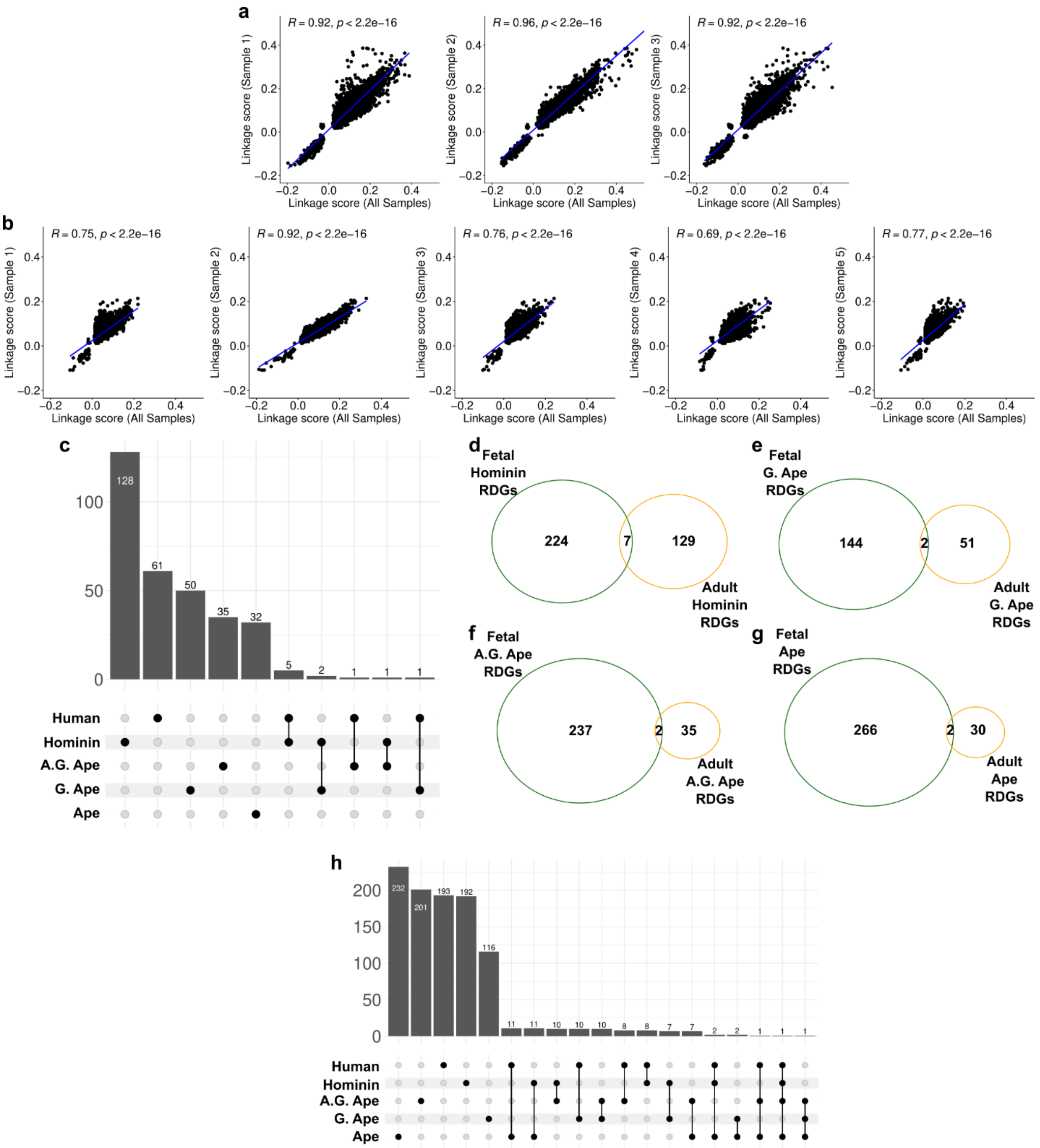

Supplementary Figure 2: Supporting analyses on regulation divergent genes. (a-b) Spearman rank correlations of gene linkage scores between biological replicates in fetal **(a)** and in adult **(b)** datasets. **(c)** Number and overlap of RDGs across lineages in adult dataset. **(d-g)** Overlap of RDGs between fetal and adult datasets per lineage. **(h)** Same as **(c)** but in fetal dataset.

bioRxiv preprint doi: https://doi.org/10.1101/2023.09.14.557820; this version posted September 17, 2023. The copyright holder for this preprint (which was not certified by peer review) is the author/funder, who has granted bioRxiv a license to display the preprint in perpetuity. It is made available under aCC-BY 4.0 International license.

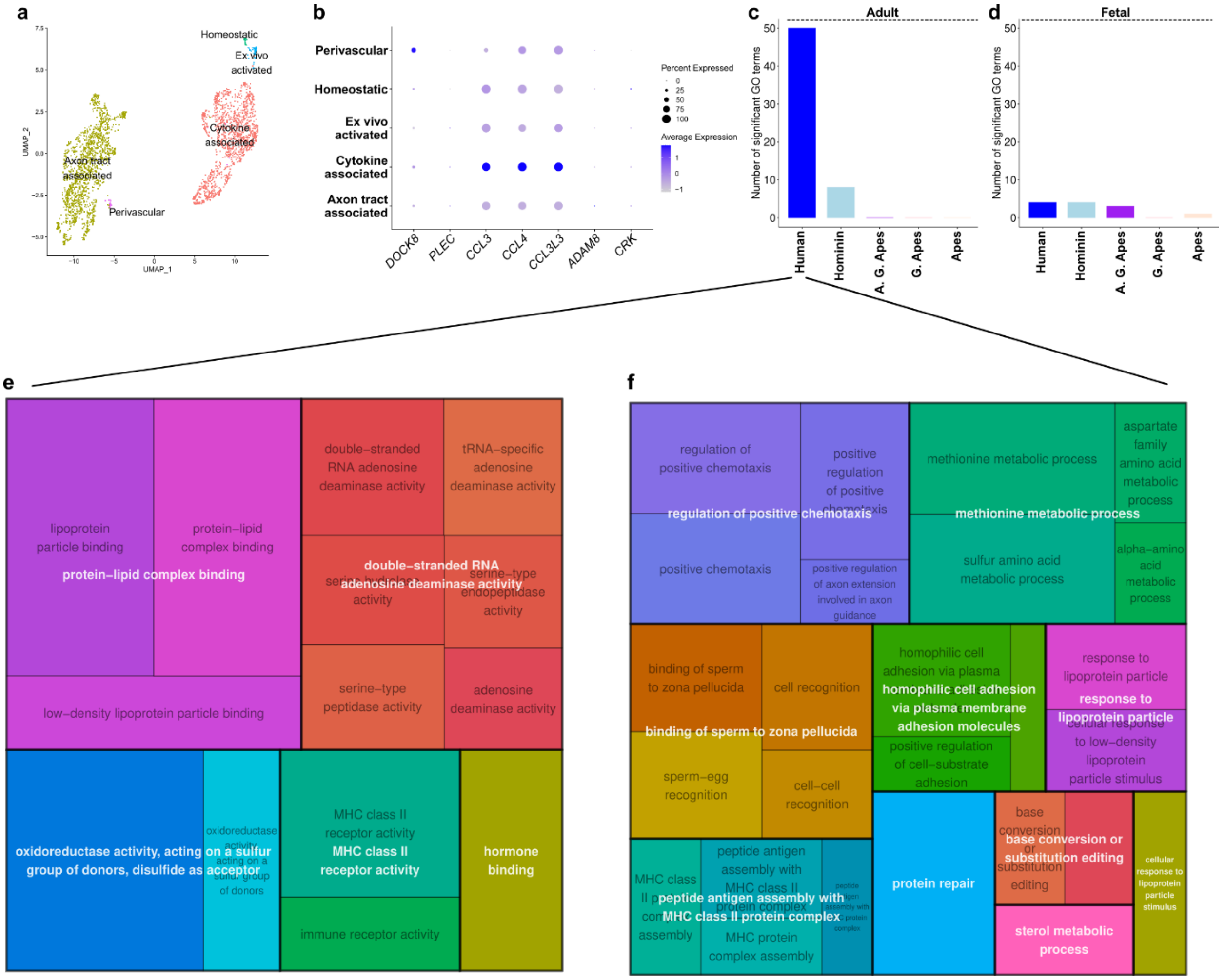

Supplementary Figure 3: (a) UMAP of annotated fetal microglial cells. **(b)** Normalized expression values of human RDGs across microglial cell types. Note that while some genes have low expression levels, these RDGs are linked to at least one microglia marker GRE and might alter expression status. **(c-d)** Number of significant GO terms associated with divergent features within each lineage. **(e-f)** Revigo plots that reduce the redundancy of human associated enrichments (molecular function **(e)** and biological process **(f)**).

bioRxiv preprint doi: https://doi.org/10.1101/2023.09.14.557820; this version posted September 17, 2023. The copyright holder for this preprint (which was not certified by peer review) is the author/funder, who has granted bioRxiv a license to display the preprint in perpetuity. It is made available under aCC-BY 4.0 International license.

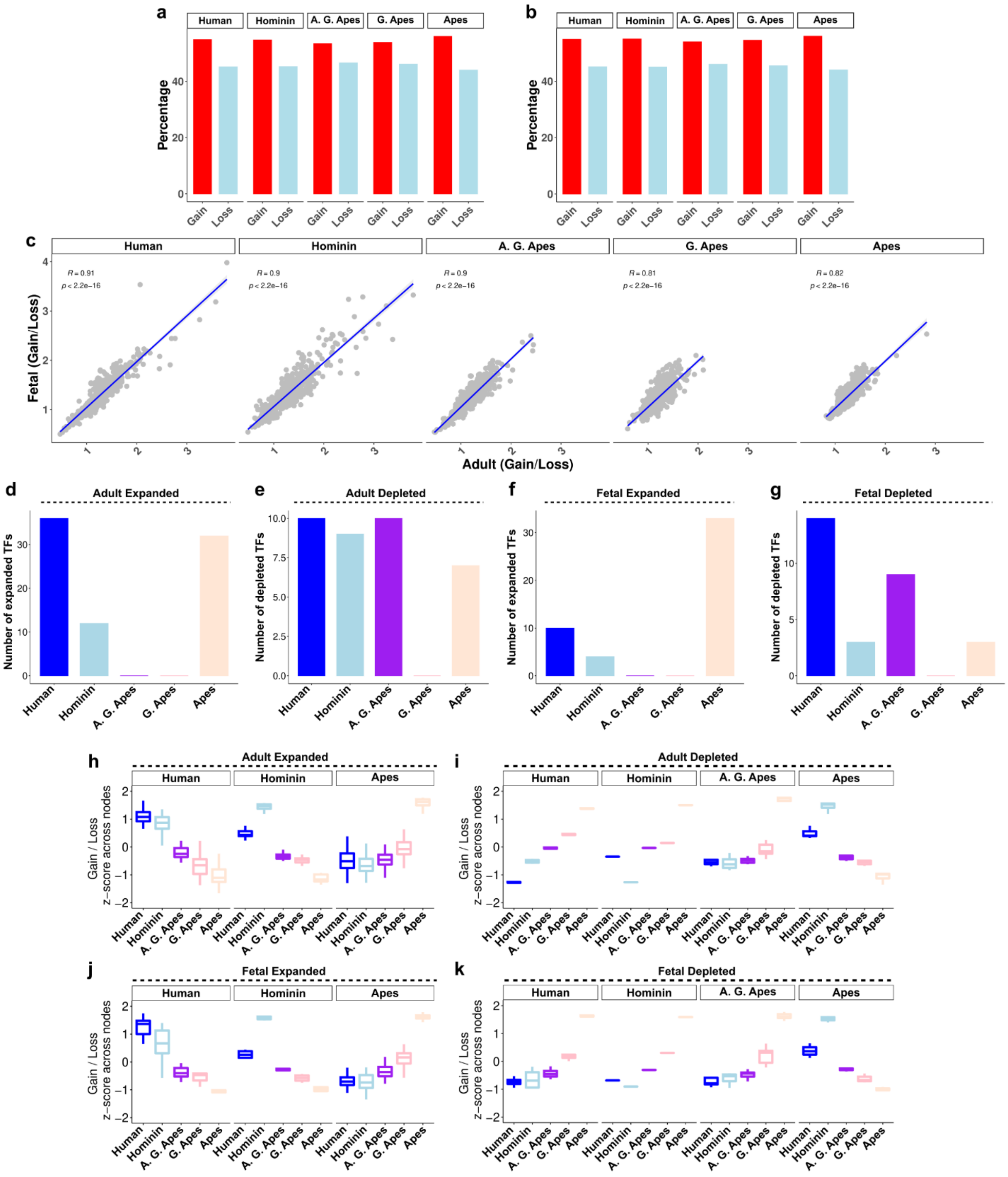

Supplementary Figure 4: Supplementary analyses on TFBS evolution. (a-b) Percentage of TFBS evolution that are gained or lost in each lineage in adult **(a)** or fetal **(b)** datasets. **(c)** Spearman rank correlations of gain / loss ratios between adult and fetal datasets per lineage. **(d-g)** Number of significantly expanded or depleted TFs per lineage in adult **(d-e)** and fetal **(f-g)** datasets. **(h-k)** Z-scores of gain / loss ratios across all lineages per TF group (expanded / depleted in adult / fetal).

bioRxiv preprint doi: https://doi.org/10.1101/2023.09.14.557820; this version posted September 17, 2023. The copyright holder for this preprint (which was not certified by peer review) is the author/funder, who has granted bioRxiv a license to display the preprint in perpetuity. It is made available under aCC-BY 4.0 International license.

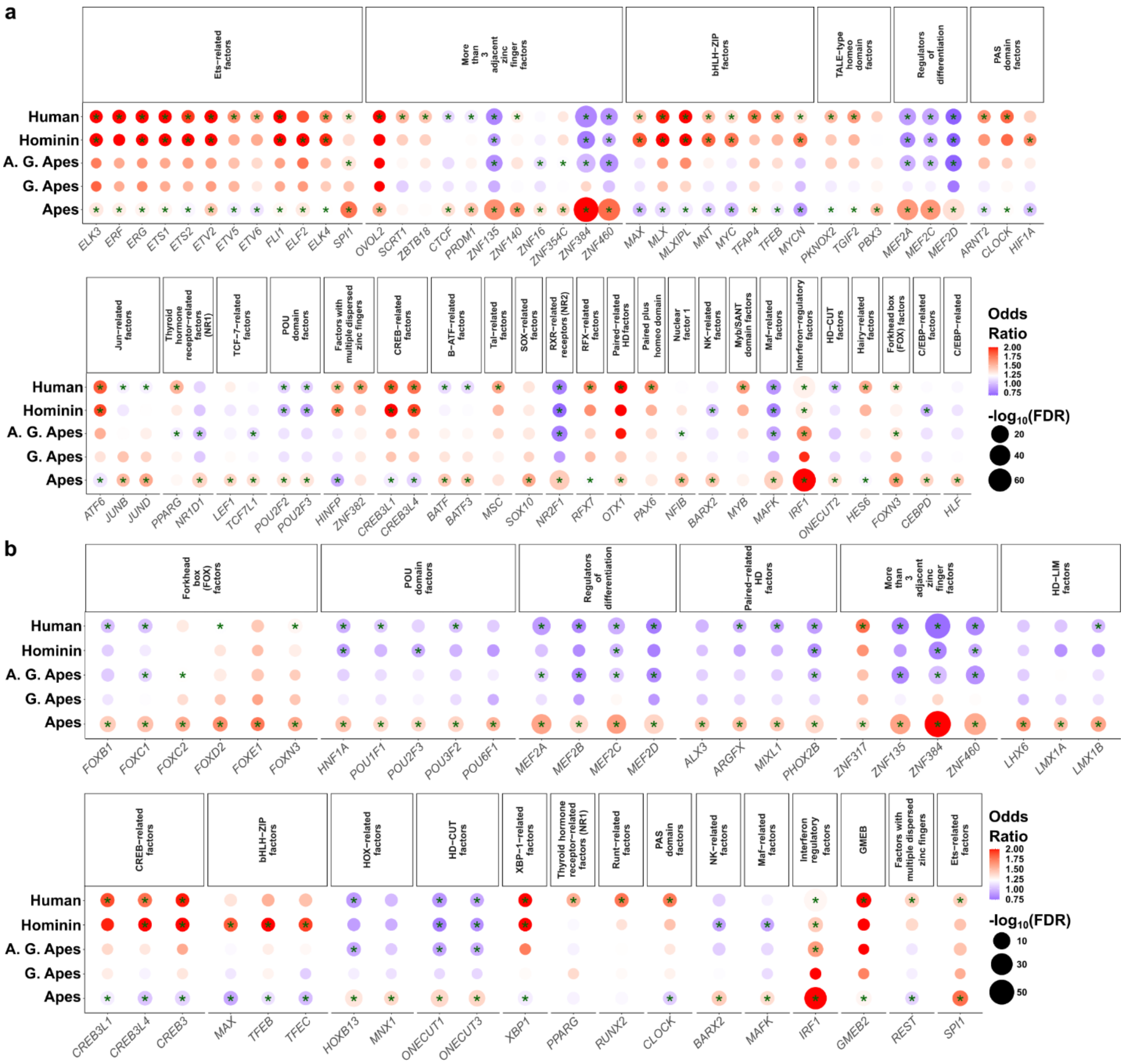

Supplementary Figure 5: List of all TFBS expansion / depletion. (a-b) TFBS expansion / depletion statistics in adult dataset **(a)** and in **(b)** fetal dataset. Asterisk indicates FDR < 0.05. Blue colors indicate depletion, red colors indicate expansion.

bioRxiv preprint doi: https://doi.org/10.1101/2023.09.14.557820; this version posted September 17, 2023. The copyright holder for this preprint (which was not certified by peer review) is the author/funder, who has granted bioRxiv a license to display the preprint in perpetuity. It is made available under aCC-BY 4.0 International license.

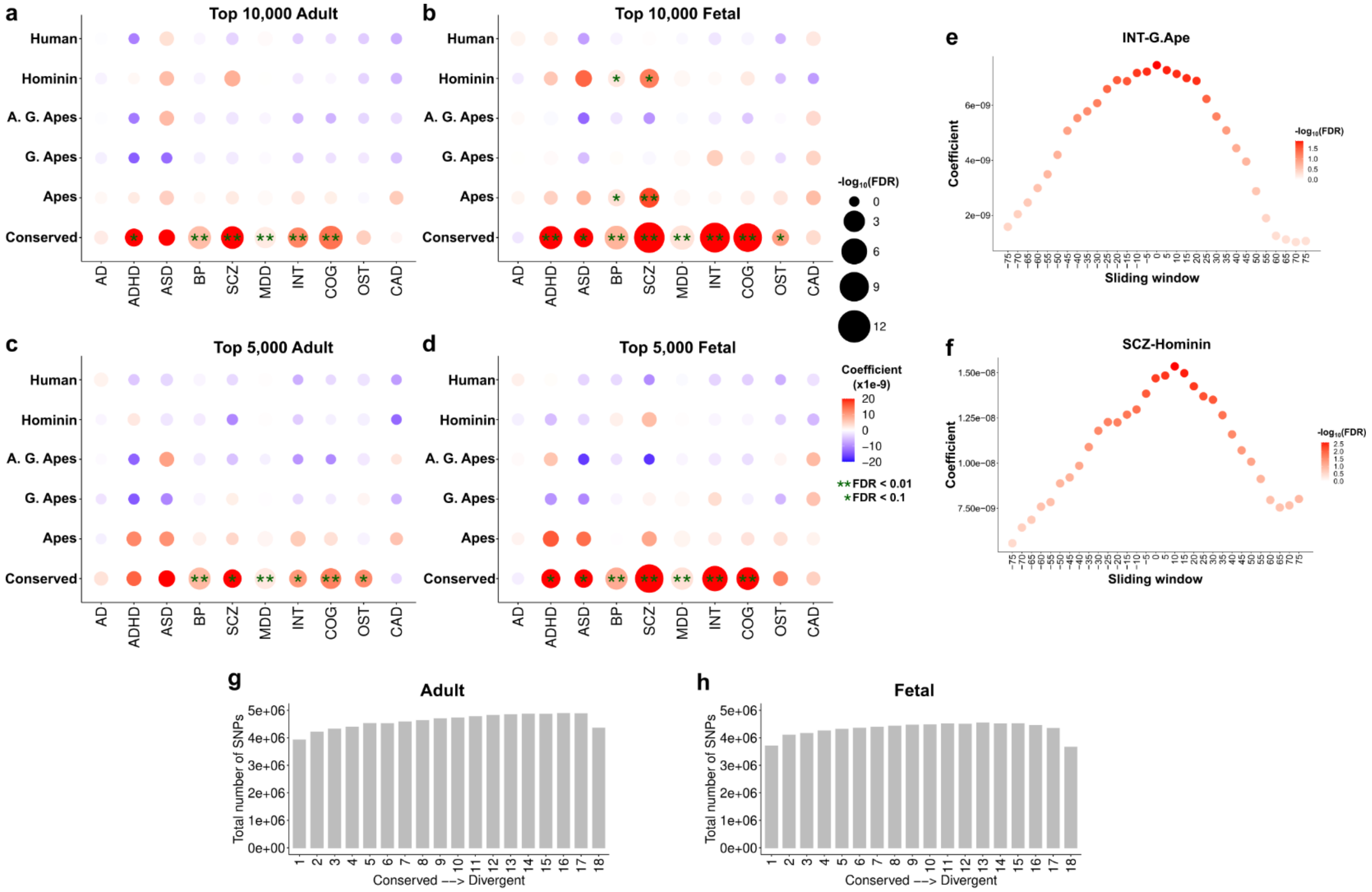

Supplementary Figure 6: Supplementary results on associations between disease variants and evolutionary divergence. (a-d) LDSC regression results between GREs grouped by evolutionary divergence (y-axis) and variants obtained from GWAS studies. **(e-f)** LDSC regressions of SCZ-Hominin and INT-G.Ape association across sliding windows. X-axis denotes the center of the 50kb window (expanded 25kb on each side). **(g-h)** Total number of SNPs mapping to the GREs grouped by conservation in adult **(g)** and fetal **(h)** datasets.

bioRxiv preprint doi: https://doi.org/10.1101/2023.09.14.557820; this version posted September 17, 2023. The copyright holder for this preprint (which was not certified by peer review) is the author/funder, who has granted bioRxiv a license to display the preprint in perpetuity. It is made available under aCC-BY 4.0 International license.

Supplementary Tables

Supplementary Table 1: Lineage-specific substitutions in adult human brain epigenome

This table provides all lineage-specific substitutions for the following lineages: Human, Hominin, A.G. Ape, G. Ape, Ape in the adult dataset.

Supplementary Table 2: Lineage-specific substitutions in fetal human brain epigenome

This table provides all lineage-specific substitutions for the following lineages: Human, Hominin, A.G. Ape, G. Ape, Ape in the fetal dataset.

Supplementary Table 3: GRE groups based on divergence and conservation

List of all lineage-divergent and conserved GREs for adult and fetal datasets. The table also includes the statistics for the criteria mentioned in the Methods.

Supplementary Table 4: Cell type marker GREs

Cell type marker GREs separately for adult and fetal datasets for the broad cell type categories mentioned in text.

Supplementary Table 5: GRE-Gene links in human brain epigenome

List of GRE-Gene links based on correlations of GRE accessibility and gene expression in multiomic single-cell adult and fetal brain datasets.

Supplementary Table 6: Regulation divergent genes (RDGs)

List of genes linked to significant excess of divergent GREs for each lineage.

Supplementary Table 7: Gene ontology enrichments

Statistics of all gene ontology enrichments.

Supplementary Table 8: TFBS gains and losses

TFBS gains and losses per lineage and per GRE.

Supplementary Table 9: TFBS expansions and depletions across lineages

List of all significant TFBS expansion and deletions across lineages.

Supplementary Table 10: LDSC statistics

All LD score regression statistics of the presented results
